# Comparative Analysis of Soil Moisture- and Weather-Based Irrigation Scheduling for Drip-Irrigated Lettuce Using Low-Cost Internet of Things Capacitive Sensors

**DOI:** 10.3390/s25051568

**Published:** 2025-03-04

**Authors:** Ahmed A. Abdelmoneim, Christa M. Al Kalaany, Giovana Dragonetti, Bilal Derardja, Roula Khadra

**Affiliations:** The Mediterranean Agronomic Institute, CIHEAM Bari, Valenzano, 70010 Bari, Italy; christamaria.kalaany@std.balamand.edu.lb (C.M.A.K.); dragonetti@iamb.it (G.D.); derardja@iamb.it (B.D.)

**Keywords:** smart irrigation, IoT, capacitive sensors, ESP32 MCU

## Abstract

Efficient irrigation management is crucial for optimizing water use and productivity in agriculture, particularly in water-scarce regions. This study evaluated the effectiveness of soil-based and weather-based irrigation management using a low-cost (DIY) Internet of Things (IoT) capacitive soil moisture sensor on drip-irrigated lettuce. A field experiment was conducted to compare water productivity and water use efficiency between the two management approaches. The soil-based system utilized real-time data from IoT sensors to guide irrigation scheduling, while the weather-based system relied on evapotranspiration data. The IoT-enabled system used 28.8% less water and reduced the pumping hours by 16.2% compared with the conventional weather-based methods. In terms of crop water productivity (CWP), the IoT system reached 16 kg/m^3^, which was 52.5% higher than the conventional method (10.5 kg/m^3^). Furthermore, the developed DIY sensor was compared with existing commercial soil moisture sensors, namely, Teros 54 and Drill& Drop. The developed prototype demonstrated reliability and accuracy comparable to other commercial sensors, with an R^2^ = 0.6, validating its utility for enhanced data-driven irrigation, giving its initial low cost (USD 62). These findings highlight the potential of low-cost soil-based IoT systems in enhancing irrigation efficiency and supporting sustainable agriculture, particularly in resource-limited settings.

## 1. Introduction

Water scarcity is an escalating global issue, intensified by climate change, population growth, and rising food demand. Agriculture accounts for over 75% of the world’s freshwater consumption, making efficient water management in this sector essential [1].

One of the main aspects that could significantly contribute toward enhanced irrigation management is reliable data acquisition systems. Such systems can provide real-time information on soil moisture, weather conditions, and plant health, which forms the backbone of precision irrigation by ensuring timely and informed decision making.

Yet, one of the primary challenges impeding the integration of such systems is the lack of cost-effective and reliable data-monitoring solutions. Given the inherent spatial variability of agricultural systems, the development and accessibility of low-cost, energy-autonomous data collection tools is essential [2,3]. Mapping spatial features is a critical step toward achieving more precise irrigation planning, real-time monitoring, and comprehensive evaluation of irrigation practices.

The integration of Internet of Things technology (IoT) into irrigation management presents a forward-thinking strategy for resource management, particularly through the implementation of smart irrigation systems. In this study, a low-cost IoT capacitive soil moisture probe was developed and used for soil-based irrigation management. The developed prototype was compared with other commercial solutions, along with comparing the resulting irrigation scheduling with weather-based irrigation scheduling to evaluate the impact of water productivity on lettuce.

Soil moisture conditions could be described using two primary terms: (i) mass-based soil water content (SWC) and (ii) energy-based soil water potential (SWP).

The soil water potential is a fundamental parameter in describing soil’s moisture condition. It plays a critical role in determining the engineering, agronomic, geological, ecological, biological, and hydrological properties of the soil mass. It could be defined as the energy that needs to be exerted by plant roots to draw water from the soil, or it could be viewed as the force exerted by the soil matrix to hold the water [4,5]. The potential energy of soil water could be represented by Equation (1):

(1) $$\psi =\psi_{m}+\psi_{o}+\psi_{p}+\psi_{g}$$

where ψ is the potential energy per unit mass, volume, or weight of water, and the sub-scripts m, o, p, and g are the matric, osmotic, pressure, and gravitational potentials, respectively [6].

The soil water content refers to the amount of moisture within a given soil sample, expressed as a ratio of either the volume or weight to the total volume or weight of the sample [7]. It provides direct information on the water content in the soil, which is crucial information for plant growth, irrigation management, and hydrological modeling. It could be described by Equation (2).

(2) $$GWC=\frac{m_{w}-m_{d}}{m_{d}}\times 100$$

where $m_{w}$ is the soil wet weight (gm), and $m_{d}$ is the soil dry weight (gm)

Two main approaches are used to measure the SWC: direct methods and indirect methods [8,9]. Direct methods involve obtaining actual soil moisture measurements through gravimetric analysis. This process entails extracting a soil sample and drying it in a laboratory oven at 105 °C for 24 h. Despite being labor-intensive, time-consuming, and destructive, this method remains the standard for soil moisture measurement due to its high accuracy [10,11,12]. However, it is unsuitable for in situ applications as it disrupts the soil structure and requires extensive manual effort.

Meanwhile, indirect methods estimate the SWC by monitoring changes in an intrinsic property of the sensing system that correlates with soil moisture variations [13]. These techniques are advantageous as they offer instantaneous, continuous, and non-destructive measurements with acceptable accuracy [14,15,16]. One of the most used indirect methods for soil moisture sensing is capacitance sensing. Capacitance sensors, in particular, have become a cornerstone in precision agriculture due to their practicality, affordability, and ease of integration into smart farming systems [17,18]. Unlike TDR and FDR, which are more expensive and complex, capacitance sensors provide a cost-effective solution for large-scale deployment [19]. This practicality has made them a favored choice for real-time soil monitoring in irrigation systems, enabling efficient water management. However, their reliance on calibration to account for soil-specific variations remains a challenge, necessitating ongoing research to improve their accuracy and applicability [20,21,22].

Capacitive soil moisture sensors are pivotal for monitoring water availability in the root zone, enabling precision irrigation. Recent advancements in capacitive sensing include the integration of IoT (Internet of Things) technologies, machine learning models for calibration, and the development of multi-sensor systems to improve measurement accuracy across heterogeneous soils [23,24]. These innovations aim to overcome the challenges associated with traditional and modern methods, providing robust solutions for soil moisture monitoring in diverse applications. With this aim, a number of studies have discussed the potential of integrating capacitive soil moisture sensors for smart irrigation management.

The authors of [22] investigated the effectiveness and accuracy of low-cost capacitive soil moisture sensors. The study found that measurements from capacitive sensors were notably affected by soil sample compaction. Specifically, variations in the output voltage of the sensors were observed. The authors of [25] designed a solar-powered capacitive soil moisture sensor and calibrated it for clay soils. The calibration results demonstrated a strong correlation between the sensor readings and soil volumetric water content, where the average determination coefficient (R2) was 0.967, while the root-mean-square error (RMSE) was 0.014.

Meanwhile, ref. [26] reported a significant variability when using capacitive soil moisture sensors. The study examined the uncertainties in the measurement process plus the natural variability in the actual soil water dynamics. Measurements were collected by 57 sensors, located at 10 combinations of depth and position relative to the drippers. The results showed large sensor-to-sensor differences, even when installed at equivalent depths and coordinates relative to the drippers. In contrast, differences between virtual sensors simulated using a HYDRUS-3D model at those soil locations were one order of magnitude smaller.

In an apple orchard trial, ref. [27] deployed 135 soil moisture sensors at 10, 20, 30, 50 and 80 cm depths. The objective of the experiment was to analyze the interplay between soil moisture dynamics and plant physiology. The study concluded that water consumption can be reduced without compromising the quality and quantity of apple production by introducing an irrigation schedule based on the information provided by capacitance sensors (ECH2O probe model EC-5).

The authors of [28] evaluated the performance of capacitive soil moisture sensors in irrigated soils, comparing them with traditional methods like gravimetric measurements. The study also highlighted the potential for capacitive sensors to improve water use efficiency by up to 15–20% when integrated into automated irrigation systems, making them a valuable tool for precision irrigation. The authors of [29] evaluated the performance of capacitance probes for monitoring soil moisture in an automated drip irrigation system for watermelons. The study found that integrating the sensors reduced water consumption by approximately 15–20% compared with conventional irrigation methods.

The authors of [30] assessed the effectiveness of capacitance probes for soil moisture monitoring in citrus orchards. The study revealed that irrigation based on the sensor data reduced water consumption by 18–22% compared with conventional irrigation methods. The probes helped maintain soil moisture at optimal levels (measuring a volumetric water content between 10 and 20%) for citrus root zones, resulting in improved growth and increased yield by 12–15% over the growing season.

Based on the reviewed literature, three main points could be concluded:

- One of the main challenges impeding the integration of smart data acquisition systems into irrigation management is the lack of cost-effective and reliable data-monitoring solutions.
- Capacitive soil moisture sensors have great potential to enhance on-farm irrigation management as they are easily integrated into smart irrigation systems.
- Yet, these sensors are mainly dependent on accurate calibration.

Within this context, this research aimed to (i) field-validate an IoT low-cost capacitive soil moisture-sensing prototype and (ii) compare soil moisture-based irrigation management using the developed prototype with weather-based irrigation management in terms of yield and water productivity under field conditions using drip-irrigated lettuce.

## 2. Materials and Methods

### 2.1. The Study Area and Experimental Setup

**Figure 1:**
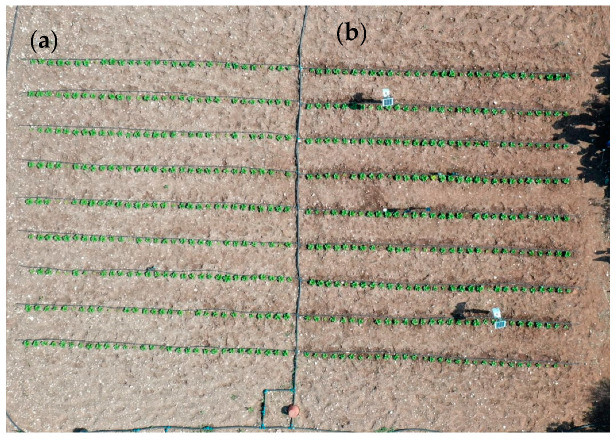
The experimental field setup: (**a**) the weather-based plot; (**b**) the soil-based plot.

The experiment was carried out at CIHEAM Bari’s experimental field in Valenzano, located in the Puglia region of southern Italy (41°2′40.3872″ N, 16°53′3.8364″ E), between 2 May and 17 June 2024. The experimental area, measuring 15 m × 9 m, was divided into two plots, each 7.5 m × 9 m. The area was transplanted with Romaine lettuce (*Lactuca sativa* L.). Both plots were equipped with a drip irrigation system, with lines spaced 1 m apart and drippers (corresponding to plant positions) spaced 0.25 m apart. The drippers were self-compensating, with a flow rate of 2 L per hour. Each 16 mm lateral feeding the drippers was fitted with a small butterfly valve to allow the precise control of individual dripper lines, a crucial feature given the differing irrigation schedules between the plots. Figure 1 illustrates the experimental setup. One of the plots was irrigated according to an irrigation schedule based on the weather data acquired from an adjacent weather station, while the other was irrigated using the installed sensors. Figure 1 shows the actual field setup using a drone image. Given the relatively small scale of the experiment (135 m^2^), the impact of the soil spatial variability on the resulting yield was neglected.

### 2.2. The Cultivated Crop (Lactuca sativa L.)

*Lactuca sativa* L., commonly known as lettuce, is a leafy green crop belonging to the Asteraceae family. It is among the most significant leafy vegetables cultivated worldwide, particularly in the Mediterranean region, where it is typically grown as a winter crop between November and February, with a total growing cycle ranging from 105 to 140 days. A second cropping cycle begins in April and lasts for approximately 75 days [31]. Being a leafy crop distinguished by its short growing cycle, it was an ideal candidate to measure the impact of irrigation-scheduling approaches on yield.

### 2.3. Plot A: Weather-Based Scheduling (Control)

In the first plot, the irrigation schedule was set based on the potential evapotranspiration (ET_0_), calculated using the Penman–Monteith method (Equation (3)). This value was adjusted using crop coefficients corresponding to different growth stages, as outlined in FAO 56, to estimate the potential crop water requirement (Equation (4)). Additional agronomic and soil parameters required to simulate lettuce growth at the site were incorporated. Table 1 provides a summary of all parameters used to develop the irrigation schedule, including their sources and whether they were estimated or measured in the laboratory.

(3) $${ET}_{0}=\frac{0.408\Delta \left(R_{n}-G\right)+\gamma \frac{900}{T+273}u_{2}(e_{s}-e_{a})}{\Delta +\gamma (1+0.34u_{2})}\pi r^{2}$$

where ET_0_ is the reference evapotranspiration (mm day^−1^), G is the soil heat flux density (MJ m^−2^ day^−1^), $R_{n}$ is the net radiation at the crop surface (MJ m^−2^ day^−1^), T is the air temperature at a 2 m height (°C), u_2_ is the wind speed at a 2 m height (m s^−1^), e_s_−e_a_ is the saturation vapor pressure deficit (kPa), e_s_ is the saturation vapor pressure (kPa), e_a_ is the actual vapor pressure (kPa), Δ is the slope vapor pressure curve (kPa °C^−1^), and γ is the psychrometric constant (kPa °C^−1^).

(4) $${ET}_{c}={ET}_{0}\cdot K_{c}$$

ET_c_ represents the potential evapotranspiration of the crop, which defines its water requirements. An effective irrigation schedule based on this model aims to replenish the ET_c_ by supplying readily available water (RAW) within the plant’s effective root zone while minimizing water losses throughout the distribution system. This could be represented by Equation (5) [15]:

(5) $$RAW=MAD\cdot \left({ϴ}_{fc}-{ϴ}_{pwp}\right)\cdot D_{r}\cdot 10$$

where MAD is the management-allowed depletion (the fraction of total available water that is allowed to be depleted before the next event), θ_fc_ is the volumetric water content at field capacity (cm^3^ · cm^−3^), θ_pwp_ is the volumetric water content at the permanent wilting point (cm^3^ · cm^−3^), and Dr is the effective root zone depth in cm. RAW is expressed in mm.

To streamline the calculation process, the AquaCrop model was employed to generate the irrigation schedule. Table 2 shows the resulting irrigation schedule. The irrigation management for this plot adhered to the generated schedule, with the daily gross irrigation requirements adjusted based on the observed rainfall during the growing season to prevent over-irrigation. The total amount of water supplied to the plot throughout the season was recorded using a water flowmeter installed upstream in the irrigation network.

AquaCrop was developed by the Food and Agriculture Organization (FAO) [33] as a tool to estimate the growth and yield of herbaceous crops, with a particular emphasis on water productivity. The program integrates key parameters, such as crop characteristics, climate data, and soil properties, to evaluate the relationship between crop performance and water availability.

### 2.4. Plot B: Soil-Based Irrigation Scheduling

Meanwhile, in the second plot, the irrigation was based on the soil water content. A threshold based on the soil water retention curve was set. For lettuce, a soil water potential between 15 and 25 Kpa is considered ideal [3,34,35]. In the experimental field soil (silty loam), this corresponded to a volumetric water content of 0.25 to 0.3 m^3^/m^3^. The objective of the irrigation schedule was to keep the soil water content within the determined thresholds using the capacitive sensors in the root zone (max.: 30 cm). Thus, only the feed from the first sensor at 15 cm was used for this scenario.

### 2.5. Design and Development of the IoT Sensors

The IoT soil moisture content probe integrated four SEN0193 capacitive soil moisture sensors with an ESP32 Lolin (WEMOS, Shenzhen, China) MCU (microcontroller unit) connected to GPIO (general purpose input/output) numbers 32, 33, 34, and 35. The prototype was enhanced with a solar-charging circuit using a TP4056 (ASIC Corp, Nanjing, China ) voltage regulator, a 2000 mAh LiPo battery, and a 2.5-Watt/5 V solar panel.

Priced at approximately USD 8–10 per unit, the SEN0193 (DFRobot, Shanghai, China) is a cost-effective choice for precision agriculture, especially when deploying multiple sensors across large fields. These sensors are coated with corrosion-resistant materials and have dimensions of approximately 98 mm × 23 mm × 3 mm (length × width × thickness), with a weight of about 15 g. Equipped with an onboard voltage regulator, they can operate within a voltage range of 3.3 to 5.5 V. The sensor includes two power pins (5 V and ground) and an analog output pin, making it well-suited for interfacing with low-voltage microcontrollers, such as the ESP32. The output is provided as a frequency signal, which ranges from 260 Hz at high moisture levels to 680 Hz at low moisture levels, where higher frequencies correspond to lower moisture contents. A built-in frequency-to-voltage converter circuit on the sensor translates the frequency signal into an output voltage that can be read as input by the microcontroller’s GPIO. The ESP32 microcontroller, featuring a 12-bit ADC (analog-to-digital converter), can represent this input voltage as an analog value within a range of 0 to 4095. In this study, the SEN0193 was lab-calibrated to the experimental field soil (silty loam) [36] to convert this analog value to its corresponding soil water content through the resulting calibration function. This was performed through extensive soil sampling, drying for 24 h at 105°, and raising the soil moisture by 10% steps while recording the resulting analog read to form the calibration curve and extract the regression function.

Meanwhile, the ESP32 Lolin is a versatile and cost-effective MCU, well-suited for IoT-based applications. It features a dual-core processor running at up to 240 MHz, 520 KB of SRAM, and integrated Wi-Fi and Bluetooth, making it ideal for real-time data collection and wireless communication. With multiple GPIO pins and a 12-bit ADC, it supports accurate analog-to-digital conversion, essential for interfacing with soil moisture sensors. One of the key advantages of the ESP32 is its deep sleep mode, which drastically minimizes power consumption, making it highly suitable for battery-powered and energy-efficient applications. While operating in active mode, the ESP32 typically draws between 95 and 240 mA, whereas in deep sleep mode, its current consumption can drop to as little as 10 µA. This remarkable reduction in energy usage, combined with its compact design, enhances its practicality for field deployments and integration into smart agriculture systems; thus, it was integrated into this prototype.

**Figure 2:**
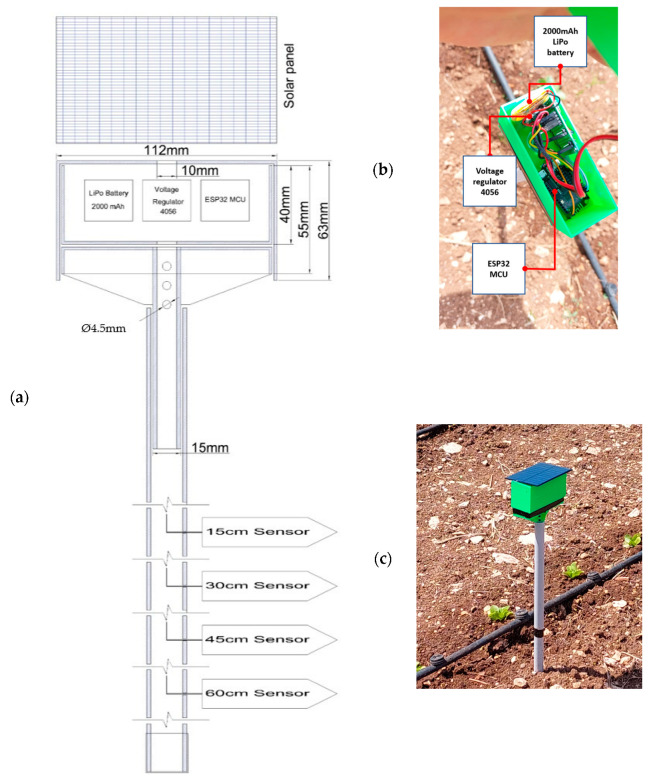
(**a**) The design of the developed prototype, (**b**) the components of the measurement unit, and (**c**) the installment in the field.

**Figure 3:**
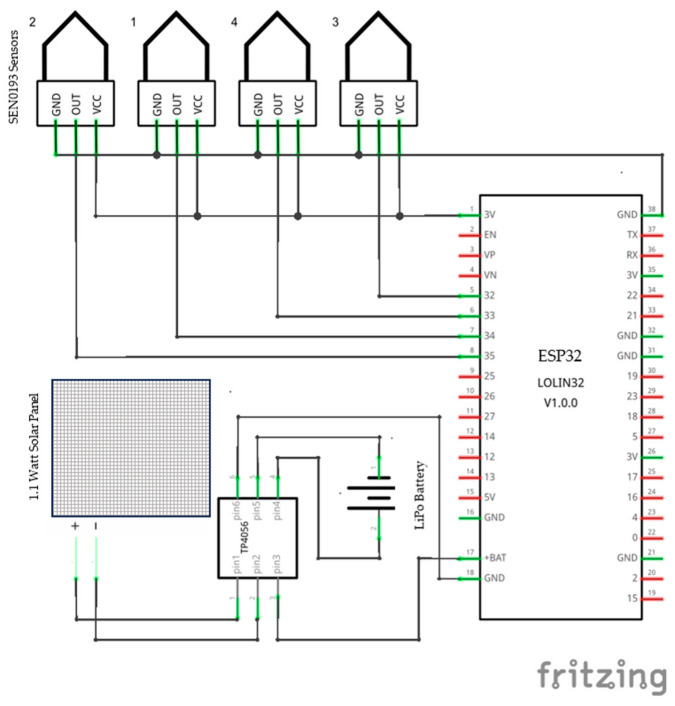
The electronics’ connection scheme.

The prototype featured an innovative and simple design, hosting the measurement system in three easy-to-3D-print parts. Figure 2a shows the design of the prototype, while Figure 2b shows the connected parts, and Figure 2c shows the final installment in the field. The first was a mount based on a 15 mm pipe, which was perforated in the upper part to allow any moisture to escape. The second was the holding box, with one lower hole for the sensor wires, while the third was a longer upper inverted box that closed tightly on the lower box and worked as a shell for the whole prototype and had an upper hole for the solar-charging wires. The solar panel was placed on the top to provide shade and protection from rain. All the diagrams and .STL files are open-source and can be replicated. The case was printed using PETG material (polyethylene terephthalate glycol), which is more tolerant to outdoor higher temperatures (up to 80–85 degrees Celsius), with 30% filling and triangular layering. This assembled measurement head was then inserted in a capped Ø20 mm, 1.2 m PVC pipe, which worked as a sleeve for the four sensor wires. The sensors were placed 15 cm apart to measure the soil moisture at 15, 30, 45, and 60 cm depths, and the wires were inserted into the PVC pipe through sealed holes up to the measurement unit, where they were soldered in a PCB (printed circuit board) connected to the MCU. The connection schematic of the prototype is shown in Figure 3. The sensors were attached to the PVC pipe using shorter cords to ensure the wires’ safety while inserting the sensors in the soil profile.

### 2.6. Algorithm Implementation

The developed prototype was capable of measuring the soil moisture content at 4 depths—15, 30, 45, and 60 cm—and sending the measured data to a cloud-based platform (ThingSpeak™) to be plotted. ThingSpeak is a cloud-based IoT analytics platform service that enables the aggregation, visualization, and analysis of real-time data streams [37].

**Figure 4:**
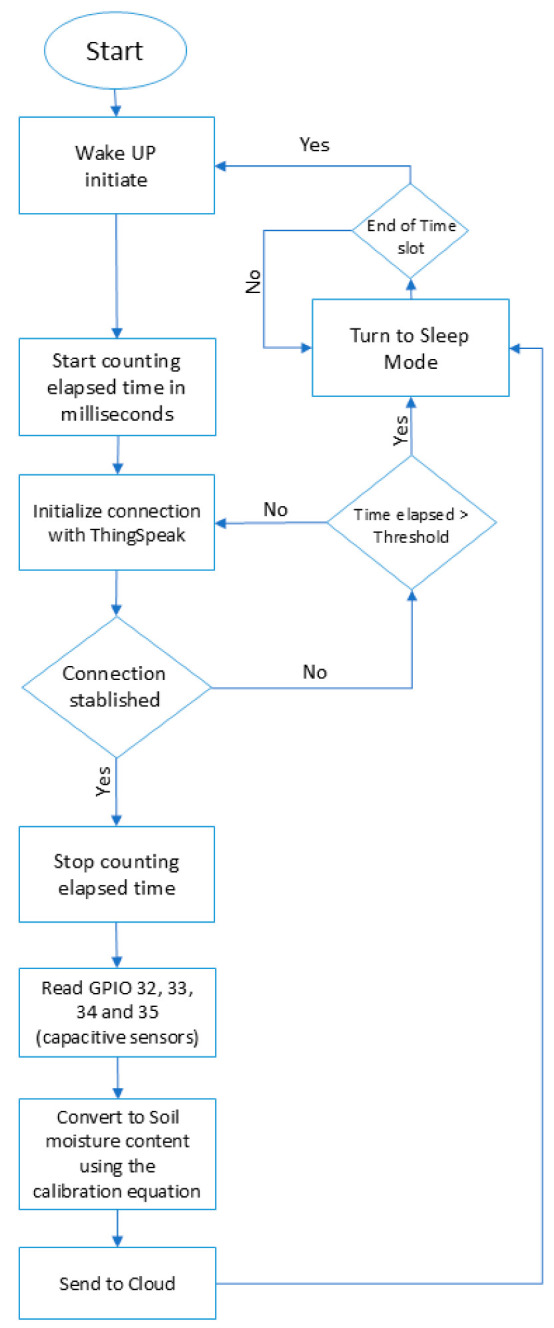
The algorithm flowchart.

Figure 4 shows how the algorithm works. Developed in C++ using the Arduino IDE, the algorithm works as follows: every six hours (referred to as the “time slot” in the flowchart), the ESP32 exits its sleep mode in an attempt to measure the soil moisture content. If successful, it transmits three data points to the ThingSpeak cloud platform using its built-in Wi-Fi module and then enters sleep mode again to conserve energy. If the microcontroller unit (MCU) cannot connect to a network within 30 s (the “threshold” indicated in the flowchart), it transitions back to sleep mode.

### 2.7. Comparison with Commercial Sensors

**Figure 5:**
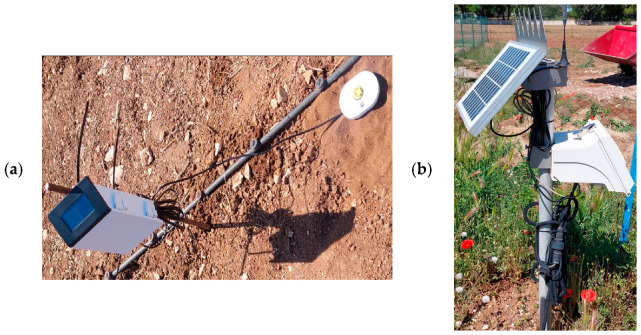
The installed commercial sensors: (**a**) TEROS 54; (**b**) Drill & Drop.

One of the secondary objectives of this study was to compare the developed soil water profile sensor with other commercial alternatives. Thus, two similar commercial soil water content probes were installed in the field. The first was a TEROS 54 (METER Group, Pullman, WA, USA) (Figure 5a), which determines the volumetric water content (VWC) of soil using capacitance-based technology. It works by identifying changes in the soil’s dielectric constant, which is a measure of its water content. It additionally considers temperature variations to ensure precise readings under diverse conditions. The sensor was positioned in the IoT plot in row 5, adjacent to the developed IoT capacitive soil moisture sensor. Meanwhile, the second was a SENTEK drill & drop (Sentek, Adelaide, Australia) (Figure 5b), which uses two electrodes that are placed in the soil to measure the changes in capacitance that take place as the soil’s water content varies, since the dielectric qualities of water are influenced by the soil’s moisture level. Two Drill & Drop sensors were placed diagonally in the IoT plot in rows 2 and 8.

## 3. Results and Discussion

The IoT capacitive probe prototype allowed for the sustainable energy-independent monitoring of the soil water content profile during the season. The data feed was uploaded to the ThingSpeak cloud service platform, enabling data-driven irrigation management. The uploaded data could be downloaded as .CSV files from the platform, enabling further analysis and archiving. Meanwhile, the weather-based field was irrigated using the irrigation schedule based on the FAO56 approach.

**Figure 6:**
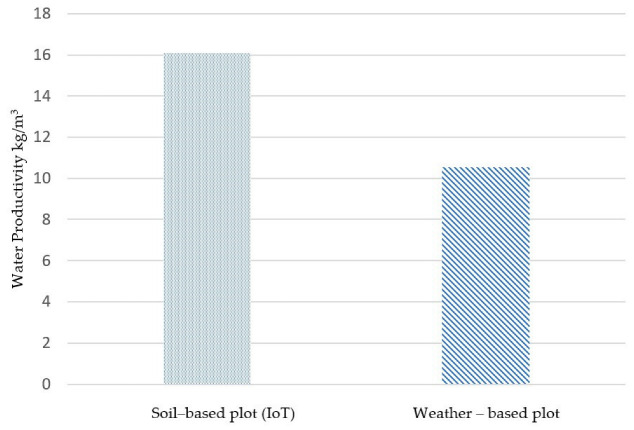
Water productivity in the IoT plot compared with the weather-based plot.

In terms of water productivity, the soil monitoring-based plot (equipped with the IoT prototype) was higher by 52% compared with the weather-based field, as shown in Figure 6. The percentage of the fresh marketable yield increment was not too high, recording 101.8 kg in the IoT plot compared with 93.8 kg in the weather-based one, yet the significant variable was the amount of water applied. In the IoT-based field, a total of 6.3 m^3^ was applied during the season compared with 8.9 m^3^ in the weather-based plot. These findings align with studies investigating soil moisture sensor-based irrigation scheduling and its impact on water use efficiency. As pointed out by [38], precision irrigation systems, while not always leading to dramatic yield increases, significantly improve water productivity, making them particularly valuable in water-scarce regions. The reduction in water consumption also aligns with [39], which reported precision irrigation systems using the IoT, and wireless sensor networks (WSNs) led to a 20–30% reduction in water usage compared with conventional methods.

Although a number of studies have investigated the use of capacitive soil moisture sensors, few have compared them to weather-based modeling, such as FAO56 based on the Penman–Monteith method [31]. The results highlight a potential overestimation of water requirements when using the FAO56 approach. Similar concerns have been raised by [40], who found that FAO56 models tend to overestimate the ET_c_, especially in semi-arid climates, which may stem from the generalized crop coefficient (Kc) and reference evapotranspiration (ET_0_) values. This finding challenges the reliance on traditional weather-based models, which, while widely used, may not account for localized soil and microclimatic conditions as effectively as IoT-based systems [41].

Additionally, soil properties play a crucial role in determining water retention and availability for plant uptake. The silty loam soil in this study, characterized by an approximately 25% stone content, contributed to lower irrigation volumes in the IoT-based system. The authors of [42] highlighted that capacitive sensors exhibit higher sensitivity to soil texture variations, which can improve precision irrigation in heterogeneous soil conditions. A similar conclusion was pointed out by [43], indicating that soil texture significantly impacts the performance of IoT-based irrigation systems. However, this also suggests that sensor calibration is crucial for accurate moisture detection, as uncalibrated capacitive sensors may yield misleading readings in soils with different hydraulic properties. Future research could explore the calibration of IoT systems for different soil types to ensure broader applicability.

The impact of soil texture on the sensors’ accuracy was also investigated by [44], where the readings of nine different sensors were compared in a silty loam soil (the same soil texture as this study). Different sensor technologies, including capacitance-based and time-domain reflectometry (TDR) sensors, exhibited varying levels of underestimation and overestimation of soil moisture content depending on the soil type. In silty loam soils, all tested sensors underestimated the ETc by 14% to 31%. This aligns with the findings of this study and highlights the importance of site-specific calibration, as [44] reported that site-specific calibration improved the soil water content estimation by 45% in silty loam and 42% in loamy sand soils, reducing error propagation into irrigation decision making.

**Figure 7:**
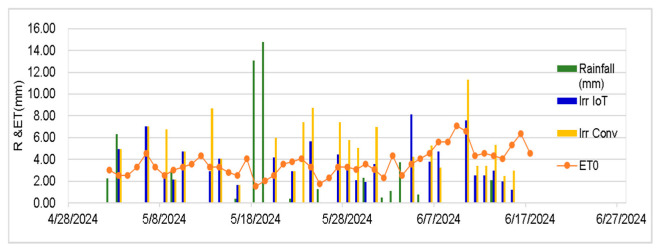
Reference evapotranspiration, rainfall, and irrigation events during the whole season in the IoT- and weather-based plots.

Figure 7 shows how the IoT-based plot was dynamically aligned with the variation in the reference evapotranspiration compared with the weather simulation scheduling. The IoT system is better positioned to optimize water usage, which could lead to more sustainable and effective agriculture activities, by directly responding to daily ET0 values. The rainfall peak was relevant for two days (18 and 19 of May 2024), with an intensive rainfall amount of 14.75 mm on 19 May and 11.33 mm on 10 June as well. Additionally, the lower irrigation volumes supplied were also due to the soil characteristics (i.e., a silt-loamy but stony soil with approximately 25% stones, lying on bedrock at 70 cm). To con clude, irrigation based on the actual soil water content fluctuation combined with IoT soil water content-monitoring systems proved to be more efficient. It is worth mentioning that the experiment was carried out on a silty loam soil, and the capacitive sensors were calibrated for it, so other soil textures with different water retention characteristics will impact the water dynamics. Additionally, variations in temperature, humidity, and evapotranspiration rates in different regions may affect the overestimation of the reference evapotranspiration, thus impacting the weather-based simulation models.

Another important advantage of the IoT system is the lower pumping hours associated with the lower irrigation applied. Although the number of irrigation events was almost the same (24 for the IoT plot and 25 for the weather-based plot), the irrigation period was lowered by 16.5%. While this suggests potential energy savings, no further investigation was conducted to measure the actual savings in energy units. Nevertheless, this was a positive initial outcome.

**Figure 8:**
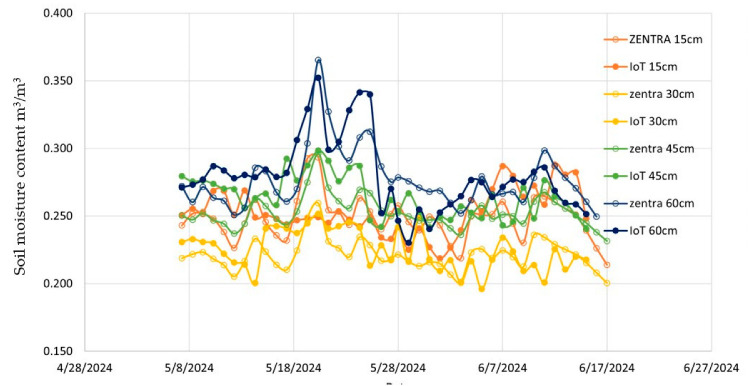
Readings from TEROS 54 and the developed prototype at four depths: 15, 30, 45, and 60 cm.

**Figure 9:**
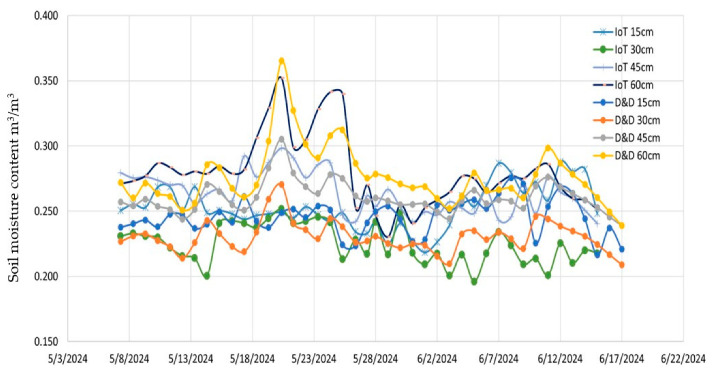
Readings from Drill & Drop and the developed prototype at four depths: 15, 30, 45, and 60 cm.

In terms of the comparison with the commercial sensors, Figure 8 shows the monitored data from the developed prototype and TEROS 54, while Figure 9 shows the same comparison with Drill and drop at 15, 30, 45, and 60 cm depths.

**Figure 10:**
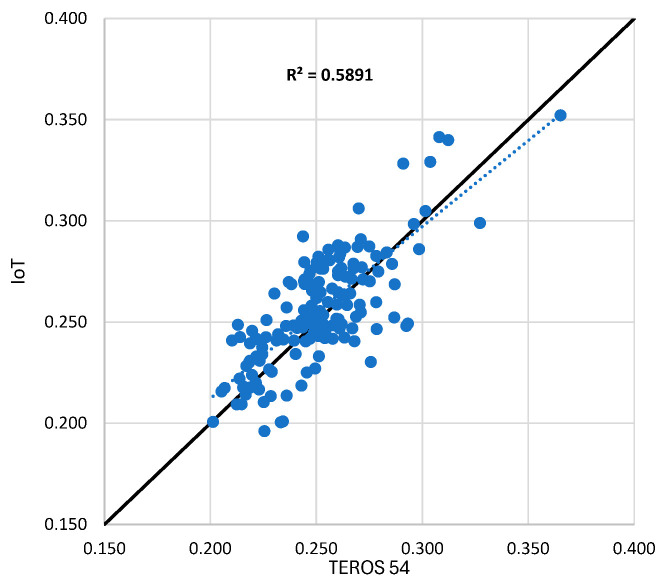
The reading from the developed prototype compared with Teros54.

**Figure 11:**
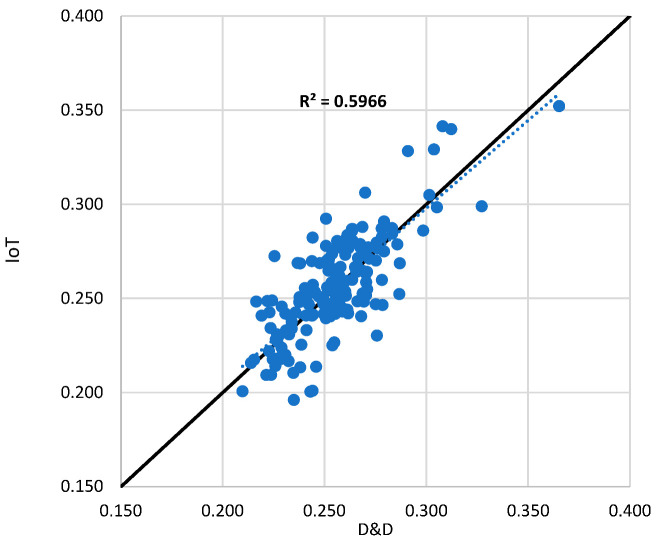
The reading from the developed prototype compared with Drill & Drop.

The developed prototype demonstrated a comparable performance, achieving a coefficient of determination (R^2^) of 0.58 when tested against the TEROS 54 and an R^2^ of 0.6 compared with the Drill & Drop sensor, as illustrated in Figure 10 and Figure 11, respectively. Notably, unlike the commercial alternatives, the prototype was specifically calibrated for the soil type used in this study, whereas commercial sensors typically rely on generalized calibration functions designed to accommodate a range of soil textures.

Nevertheless, this prototype is very cost-effective compared with the commercial sensors. Table 3 shows the actual costs of the DIY prototype. Although this cost is not comparable to the actual market prices of commercial products, as it is a DIY prototype, the alternatives are around 10 times higher, ranging between USD 800 and 1500. With comparable performance, this developed open-source DIY soil moisture-monitoring system proved to be reliable, efficient, and low-cost for smart on-farm irrigation management. An equally important factor is the absence of subscription fees typically required by service providers to access data via their platforms. Open-source, DIY prototypes like the one presented here provide cost-effective, innovative solutions while eliminating the constraints of data management fees and service provider ownership of data.

## 4. Conclusions

This study demonstrated that integrating low-cost, IoT-based capacitive soil moisture sensors into irrigation management can significantly enhance water productivity. The IoT-enabled system reduced water application by 28.8% compared with conventional weather-based methods while achieving a higher yield of 101.8 kg, surpassing the 93.8 kg yield under traditional practices. Consequently, the crop water productivity (CWP) of the IoT system reached 16 kg/m^3^, a 52.5% improvement over the conventional method’s 10.5 kg/m^3^. The developed sensor was constructed from affordable and readily available components, with a total cost of approximately USD 62, making it accessible for smallholder farmers and resource-limited agricultural settings. Beyond water efficiency, this study highlighted potential energy savings, as the IoT-based system reduced pumping hours by 16.5% despite a similar number of irrigation events, suggesting additional benefits in lowering energy consumption and operational costs for farmers. The affordability, reliability, and efficiency of this open-source IoT-driven irrigation system emphasize its potential. By enabling sustainable water management, minimizing the environmental impact, and supporting long-term resource conservation, IoT-enabled precision irrigation systems can play a pivotal role in building resilient and efficient agricultural production systems in the face of climate change and water scarcity.

## Figures and Tables

**Table 1:** Climatic, agronomic, and soil parameters used to generate the weather-based irrigation schedule.

Parameter		Reference/Source	
Rainfall (mm)	Daily data for the past 3 years provided by the weather station situated close to the field at CIHEAM Bari		
Evapotranspiration (mm)			
Minimum and maximum Temperature (°C)			
Mean annual CO_2_ concentration (ppm)	MaunaLoa.CO_2_ file from AquaCrop database		
Calendar	Growing period	From 21 April to 21 June 2024	
Crop	Description	Display crop parameters: full set	
	Development	Initial canopy cover: 2.25% Type of planting method: transplanting Maximum canopy cover: 60 days after transplant Root deepening: shallow-rooted crop (max.: 0.30) Canopy growth coefficient (CGC): 15%/days	[32]
	Fertility stress	Not considered	
	Salinity and cold stress		
	Temperature	Base temperature for crop development: 7 °C Upper temperature for crop development: 30 °C	
	Water	Canopy expansion: moderately tolerant water stress Upper threshold for canopy expansion: 0.25 Lower threshold for canopy expansion: 0.55 Shape factor for stress coefficient of canopy expansion Stomatal closure: moderately sensitive to water stress Upper threshold for canopy expansion: 0.50 Shape factor for stress coefficient for stomatal closure: 3	
	Type	Annuals: leafy vegetable crops Type of photosynthetic pathway: C3 crop	From the FAO irrigation and drainage paper No.56 “Crop evapotranspiration”
Irrigation	Mode	Generation of irrigation schedule	Chosen by user preferences
	Irrigation method	Drip irrigation Percentage of soil surface wetted: 30%	
	Time and depth criteria	Time criteria: allowable depletion (20% of RAW) Depth criteria: back to field capacity Irrigation water quality: excellent	
Field	None		
Soil profile	Characteristics of soil horizons	Description: silty loam (clay (17.25%), silt (59.25%), and sand (23.5)) Thickness: 1.20 m TAW: 130 mm/m PWP: 13 vol% FC: 26.0 vol% SAT: 46 vol% Hydraulic conductivity: 150 mm/day	Measured through soil texture and structure analyses, performed in the CIHEAM Bari soil lab
Groundwater	None		

**Table 2:** Irrigation schedule generated by AquaCrop model for the irrigation of the control plot.

Date	Net Application (mm)
2 May	9
8 May	8
12 May	5.3
18 May	4.9
23 May	5.9
25 May	7.3
27 May	4.8
29 May	5
1 June	4.6
3 June	6.9
9 June	5.4
11 June	7.2
13 June	6.9
17 June	4.8
18 June	4.1
19 June	4
20 June	4.1
22 June	5.5
23 June	6.2
24 June	4.8
27 June	9.2
28 June	5
29 June	4.1
30 June	4.3
2 July	7.2

**Table 3:** Breakdown of the cost of the prototype.

Item	Quantity	Cost (USD)
ESP32 Lolin	1	10
SEN0193	4	11.5
MP4056	1	5.5
PVC pipe	2 m	2.25
Rubber cap	1	3.20
2000 mAh LiPo battery	1	8
2.5 W, 5 V solar panel	1	8
Miscellaneous (wires, isolation tape, pins, etc.)	1	10
PETG filament	200 gm	3.5
Total		61.95

## Data Availability

The data presented in this study are available upon request from the corresponding author.
